# Radiation‐induced myocardial fibrosis: Mechanisms underlying its pathogenesis and therapeutic strategies

**DOI:** 10.1111/jcmm.15479

**Published:** 2020-06-14

**Authors:** Bin Wang, Huanhuan Wang, Mengmeng Zhang, Rui Ji, Jinlong Wei, Ying Xin, Xin Jiang

**Affiliations:** ^1^ Department of Radiation Oncology The First Hospital of Jilin University Changchun China; ^2^ Jilin Provincial Key Laboratory of Radiation Oncology & Therapy The First Hospital of Jilin University Changchun China; ^3^ NHC Key Laboratory of Radiobiology School of Public Health Jilin University Changchun China; ^4^ Phase I Clinical Research Center The First Hospital of Jilin University Changchun China; ^5^ Department of Biology Valencia College Orlando FL USA; ^6^ Key Laboratory of Pathobiology Ministry of Education Jilin University Changchun China

**Keywords:** micro‐RNAs, radiation‐induced myocardial fibrosis, reactive oxygen species, therapeutic strategies, transforming growth factor β1

## Abstract

Radiation‐induced myocardial fibrosis (RIMF) is a potentially lethal clinical complication of chest radiotherapy (RT) and a final stage of radiation‐induced heart disease (RIHD). RIMF is characterized by decreased ventricular elasticity and distensibility, which can result in decreased ejection fraction, heart failure and even sudden cardiac death. Together, these conditions impair the long‐term health of post‐RT survivors and limit the dose and intensity of RT required to effectively kill tumour cells. Although the exact mechanisms involving in RIMF are unclear, increasing evidence indicates that the occurrence of RIMF is related to various cells, regulatory molecules and cytokines. However, accurately diagnosing and identifying patients who may progress to RIMF has been challenging. Despite the urgent need for an effective treatment, there is currently no medical therapy for RIMF approved for routine clinical application. In this review, we investigated the underlying pathophysiology involved in the initiation and progression of RIMF before outlining potential preventative and therapeutic strategies to counter this toxicity.

## 1 INTRODUCTION

In recent decades, chest RT has been an effective part of clinical multimodality therapy for breast cancer, Hodgkin's lymphoma, oesophageal cancer, lung cancer and other malignancies involving the intrathoracic and chest wall regions.^1^ It has been estimated that more than half of patients with these conditions receive RT at some point in time.^2^ However, exposure of all or part of the heart during RT poses an increased risk of RIHD and has become an important clinical concern for the growing patient population.^2^ Previous research has found that cardiac toxicity after RT is the leading cause of non‐cancer‐related mortality.^3^ RIHD can involve any part of the heart, from subclinical histopathologic changes to obvious clinical disease such as conduction system abnormalities, cardiomyopathy, pericarditis, valvular heart disease, coronary artery disease and myocardial fibrosis.^4^ RIMF is a major stage of RIHD, which has an incidence as high as 20%–80%.^5^ The development of RIMF is a slow but constantly progressive process, with clinical symptoms occurring several years after irradiation. RIMF increases myocardial stiffness, decreases myocardial systolic and diastolic function, and causes perfusion and ejection fraction defects leading to myocardial electrophysiological disorders, arrhythmias, heart failure or even sudden death. Together, these sequelae impair the long‐term health of post‐RT survivors and limit the dose and intensity of RT required to effectively kill tumour cells.^6^

It has been clearly established that the incidence and severity of RIMF may be aggravated by its association with a number of risk factors; these include the volume and region of irradiated heart, the total dose and dose per fraction/target volume ratio, time since exposure, age at exposure, metabolic factors such as diabetes, hypertension, dyslipidaemia and obesity, smoking, and combination therapy with cytotoxic chemotherapy like anthracyclines, trastuzumab or endocrine therapy.^7^ Given that the presence of conventional risk factors potentiates RIMF, aggressive risk factor management should ideally be initiated prior to RT. Assessment and diagnosis of RIMF are often challenging and usually result in a diagnosis of exclusion due to the long latency period between radiation exposure and the development of symptomatic RIMF.^2^ Therefore, studies with RIMF as an end‐point may be helpful for the early prevention and diagnosis of radiation‐induced cardiotoxicity.

As the underlying development of RIMF remains poorly understood, there is no effective clinical treatment for the condition and it continues to impair the quality of life of long‐term cancer survivors. Therefore, new strategies that can stop or even reverse the course of RIMF are urgently needed. In this review, we describe the underlying pathophysiology of RIMF and outline potential preventative and therapeutic strategies to counter this toxicity, providing references for the management of RIMF.

## 2 POTENTIAL EVENTS INVOLVED IN THE PATHOGENESIS OF RIMF

**FIGURE 1:**
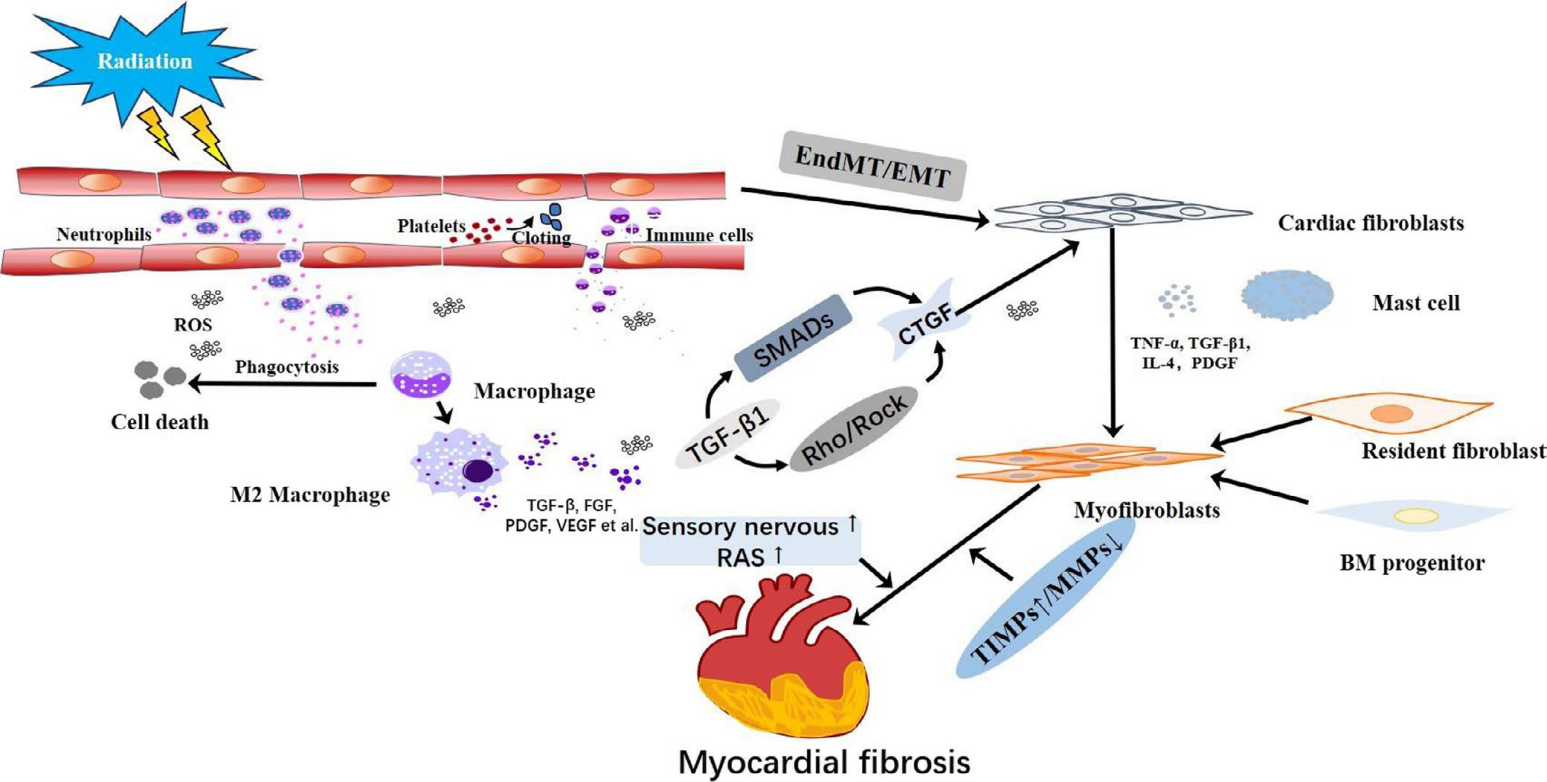
The pathogenic mechanisms of RIMF

Although the exact mechanisms involved in RIMF pathogenesis are unclear, myocardial fibrosis after irradiation seems to be a lengthy process of multicellular interactions mediated by multiple contributory events. Below, we shed light on the current understanding of the pathogenic mechanisms of RIMF obtained from preclinical studies (Figure 1).

### 2.1 Vascular event: vascular injury and endothelial dysfunction

Data from human and animal studies have indicated that vascular injury and endothelial dysfunction play a pivotal role in the development of RIMF. The myocardial subunit is composed of cardiomyocytes, stromal tissue and capillaries. Lacking arterioles in the tissue, each myocardial subunit has a capillary network that relies on diffusion for the exchange of nutrients and metabolites.^8^ While cardiomyocytes are relatively radiation‐resistant because of their slow mitotic rate, the abundant myocardial capillary network remains the main radiation damage target for the intrinsic sensitivity of endothelial cells.^9^ Endothelial dysfunction leading to pro‐inflammatory and pro‐fibrotic environments is a common aspect of radiation injury in normal tissue. Within minutes of ionizing radiation (IR) exposure, endothelial dysfunction causes the excessive production of eicosanoids such as leukotrienes, thromboxane, prostacyclin and prostaglandins, leading to vasodilation, increased capillary wall permeability and leucocyte extravasation.^10^

Some evidence has shown that the imbalance between reactive oxygen species (ROS) and nitric oxide (NO) is responsible for vascular injury after irradiation. After vascular endothelial cells are exposure to IR, NO (a vascular protectant) can be directly scavenged by rapid production of ROS, which cause the formation of peroxynitrite to initiate nitrosylation of tyrosine residues of proteins and lipid peroxidation.^11^ This eventually thought to result in impaired vasomotor responses and vessel stenosis.^11^, ^12^ Up‐regulation of NADPH oxidases (NOXs) after irradiation, especially NOX2 and NOX4 that are abundantly expressed in vascular endothelial cells and cardiomyocytes, can promote the overproduction of oxygen free radicals and induce redox reactions, altering calcium homeostasis and disordering metabolism of vascular endothelium.^13^, ^14^, ^15^ Evidence from rat models indicates a reduction of alkaline phosphatase (ALP) in capillary endothelial cells within a few weeks of heart irradiation. ALP is abundant in healthy cardiac microvasculature and its loss is a particularly sensitive sign of endothelial cell injury.^10^ Preceded by increased endothelial proliferation, capillary loss only occurs in enzyme‐negative areas. 16Sub‐endothelial extracellular matrix (ECM) components exposed to platelets after radiation can promote the secretion of von Willebrand factor and decrease the secretion of adenosine diphosphatase and thrombomodulin; these factors trigger an antifibrinolytic‐coagulation cascade effect, causing blood coagulation and vascular occlusion.^17^ Although the remaining capillary endothelial cells respond to injury by increasing proliferation, this mechanism is inadequate for maintaining proper microvascular function.^18^ In one animal study, focal myocardial degeneration was found to occur in the centre of the foci of capillary loss.^19^ Furthermore, the progressive decrease of capillary density eventually results in ischaemia, cardiomyocyte apoptosis and fibrosis.^20^ Chronic hypoxia caused by microvascular injury may also up‐regulate the expression of hypoxia‐inducible factor 1α (HIF‐1α).^21^ HIF‐1α is a proven powerful stimulator of various pro‐fibrotic mediators such as transforming growth factor‐β (TGF‐β), endothelin‐1 (ET‐1), vascular endothelial growth factor (VEGF), as well as connective tissue growth factor (CTGF), which directly or indirectly facilitates fibrosis.^21^, ^22^ It has been confirmed that HIF‐1α may cause fibrin accumulation by augmenting plasminogen activator inhibitor‐1.^23^ Chronic hypoxia and the expression of HIF‐1α can trigger angiogenesis by up‐regulating the expression of VEGF and TGF‐β.^24^ Besides, hypoxia can also induce hematopoietic stem cells to homing to tumour increasing cancer cell survival through TGF‐β‐HIF‐1α pathway and the induction of inflammatory mediators like cyclooxygenase‐2 (COX‐2).^25^, ^26^

IR can also cause damage to the coronary arteries and accelerate the development of coronary atherosclerosis. It is known that injury and senescence of endothelial cells lining the major arteries are essential for the initiation and development of atherosclerosis. The initial event of coronary atherosclerosis after irradiation involves endothelial cell injury and the migration of monocytes to the intima. Experimental studies have shown that doses ≥ 2 Gy can induce the expression of inflammatory adhesion molecules and improve the adhesion of leucocytes. Damaged endothelial cells increase the capacity for monocyte adhesion through inflammatory adhesion molecules such as vascular cell adhesion molecule 1 (VCAM‐1), intercellular adhesion molecule‐1 (ICAM‐1) and E‐selectin.^27^ Invading monocytes are transformed into activated macrophages and recruited into the intima via monocyte chemotactic protein‐1 to form fatty streaks in the presence of elevated cholesterol.^27^ Monocytes absorb low‐density lipoprotein to form lipid‐laden foam cell aggregates after transmigration, which further secrete inflammatory cytokines and pro‐fibrotic chemokines (ie TGF‐β), stimulate the differentiation of smooth muscle cells into myofibroblasts, and generate large quantities of type IV collagen, eventually leading to arterial stenosis.^28^ The formation of plaque after irradiation may be more prone to intraplaque haemorrhage and rupture, with significantly increased levels of granulocytes and macrophages.^29^ The mid‐ and distal left anterior descending branch of arteries is mainly involved in RIMF.^30^

### 2.2 Oxidating event: ROS‐mediated oxidative stress

Under normal physiological conditions, physiological levels of intracellular ROS, such as hydrogen peroxide, nitric oxide, superoxide and peroxynitrite, are considered major mediators of homeostasis maintenance, cell proliferation, cell differentiation, immune responses, proper functioning of endothelial cells and myocyte contraction.^31^ The electron‐transport chain (ETC) in mitochondrion is the major source of ROS, IR can cause mitochondrial malfunction and change the activity of the ETC complexes/oxidative phosphorylation, leading to an overproduction of superoxide containing ROS and cardiomyocyte apoptosis.^33^ IR can also cause an abnormal increase in pro‐oxidant enzymes (mainly NADPH oxidases) and the destruction of endogenous antioxidants (including superoxide dismutase, glutathione peroxide and catalase), resulting in sustained ROS generation.^32^, ^34^ NADPH oxidases have seven subfamilies including NOX1‐5 as well as two dual oxidases (DUOX1 and DUOX2), which are effective enzymatic source of ROS.^35^ The activities of these enzymes are extensively regulated by various growth factors and cytokines like TGF‐β and interleukins (eg IL‐1, IL‐4 and IL‐13), which are powerful inducers of fibrosis by inducing epithelial‐mesenchymal transition (EMT), the production of fibronectin and α‐smooth muscle actin (a‐SMA) and the up‐regulation of collagen 1 and 3.^33^ Up‐regulation of NOX1 in the endothelial cells in response to IR can enhance differentiation of fibroblasts, inhibition of which significantly decreases collagen deposition and fibroblastic changes.^36^ Up‐regulation of NOX2 and NOX4 expressed abundantly in cardiomyocytes and endothelial cells after exposure to IR can trigger the process of ROS burst mediating cardiomyocyte death and myocardial fibrosis.^14^, ^35^ And NOX2 and NOX4 may participate in radiation‐induced bystander effect, leading to the generation of ROS in nearby non‐irradiated cells.^25^ NOX4 and NOX5 have been shown to mediate chronic oxidative stress (OS) of human fibroblast cells. Using antioxidants to inactivate these enzymes can decrease the expression of TGF‐β and ECM accumulation.^37^, ^38^

Increased expression of both DUOX1/DUOX2 in tissues like heart and lung has been observed across some studies.^32^ Up‐regulation of DUOX1/DUOX2 after irradiation may lead to chronic oxidative damage and infiltration of inflammatory cells like lymphocytes and macrophages, which can undermine myocardial blood supply and lead to hypertrophy and fibrosis.^26^, ^35^ It has been confirmed that the activation of IL‐13 induced by p38 mitogen‐activated protein kinases (MAPK) after irradiation can increase DUOX1 expression, resulting in sustained ROS production and continuous DNA damage.^38^ Data from rat models suggest that increased the level of DUOX1 and DUOX2 was obviously up‐regulated following an increase in IL‐4 after exposure to IR, thus inducing ROS generation ^39^, ^40^ Several radioprotectors and antioxidants like metformin, melatonin and selenium have been proved to suppress up‐regulation of these genes including DUOX1/DUOX 2 and pathways of IL‐4 or IL‐13, thereby alleviating damage to tissues of heart and lung induced by radiation.^40^, ^41^, ^42^ In addition, NOX1‐5 and DUOX1/DUOX2 can amplify OS in a positive feedback loop by triggering other redox mediators, which stimulate more release of pro‐inflammatory and pro‐fibrotic mediators.^14^, ^35^

Several researches have indicated that increased COX‐2 expression after irradiation exposure is closely related to vascular injury, atherosclerosis and fibrosis in heart tissue.^32^, ^43^ COX‐2 is one of the three isoforms of cyclooxygenase, generating prostaglandins (PGs) by arachidonic acid metabolism.^15^ ROS is the standard secondary metabolites in the synthesis of PGE2 through COX‐2. Increased expression of COX‐2 following DNA damage and cell death induced by IR can amplify ROS production, resulting in continuous oxidative damage and inflammation responses.^44^ The strong catalytic effect of TGF‐β pathway on COX‐2 gene expression has been confirmed.^25^ TGF‐β and TGF‐βRI can augment COX‐2 expression through non‐canonical pathway and canonical pathway, respectively.^45^, ^46^

In the cardiovascular system, high concentrations of ROS play a pathophysiological role in the processes of inflammation, hypertrophy, apoptosis, proliferation, migration, fibrosis and angiogenesis, which in turn contribute to hypertension, atherosclerosis and heart failure. ROS can promote the initiation and perpetuation of the pro‐fibrotic process mediated by TGF‐β1. Furthermore, ROS can promote the release of calcium from the endoplasmic reticulum, which causes calcium overload in the mitochondria and leads to membrane swelling and the release of apoptotic factors; together, these processes increase the generation of ROS.^4^ ROS can also alter the expression of various proteomes in the cytoplasm and can simultaneously increase adhesion molecules, inflammatory mediators and proteases, while decreasing NO and blocking the aggregation of platelets and the proliferation of vascular smooth muscle.^47^ As a second messenger signal in cells, ROS participates in the regulation of signalling pathways, including MAPK and nuclear factor‐κB (NF‐κB). NF‐κB is a protein complex that regulates DNA transcription involved in cellular stress responses and synchronizes the expression of chemokines, cytokines and adhesion molecules in endothelial cells. This mechanism may serve as a key link between OS and several inflammatory pathways.^48^ There is evidence that excessive production of ROS and other free radicals can mediate epigenetic regulation in fibroblasts by targeting DNA methylation, histone methylation and acetylation to induce the differentiation of fibroblasts to myofibroblasts.^32^

### 2.3 Inflammatory event: continuous inflammatory processes

It is generally believed that the early pro‐inflammatory environment at exposure sites proximal to the vasculature is a strong initiator of myocardial fibrosis.^49^ Shortly after exposure, swelling and apoptosis of vascular endothelial cells induce inflammatory chemokine secretion and degradation of the endothelial basement membrane by matrix metalloproteinases (MMPs) to recruit leucocytes to injured sites.^50^, ^51^ Neutrophils first adhere to the endothelium and migrate to damaged sites, mediated by adhesion molecules like E‐selectin, ICAM‐1 and VCAM‐1.^52^ Neutrophils then secrete growth factors and inflammatory mediators including tumour necrosis factor (TNF), IL‐1, IL‐6 and IL‐8, to mediate the acute inflammation response.^53^ While several mediators promote the recruitment of inflammatory and pro‐fibrotic cells, others like IL‐1 mediate radioprotective effects.^50^ Within hours of exposure, pro‐fibrotic cytokines like platelet‐derived growth factor (PDGF), insulin‐like growth factor (IGF), basic fibroblast growth factor (FGF), TGF‐β and CTGF are released to promote ROS development, which in turn lead to chronic inflammation.^21^ Endothelial inflammation may adversely migrate to recipient cells through the secretome via the signal transducer and activator of transcription (STAT)‐mediated process.^54^ Subsequently, interactions between monocytes and lymphocytes promote the differentiation of monocytes into two macrophage subsets—classically activated M1 macrophages and alternatively activated M2 macrophages. M2 macrophages can produce PDGF and VEGF to stimulate neoangiogenesis and migrate fibroblasts to the damaged site. Increased M2 macrophages also enhance proliferation and differentiation of fibroblasts into myofibroblasts by the secretion of TGF‐β and FGF.^55^ A variety of mediators eventually lead to the continuos recruitment of MMPs and inflammatory cytokines, including TGF‐β, IL‐4 and IL‐13, as well as the proliferation of smooth muscle cells. IL‐13 secreted by inflammatory T cells is gradually recognized as a potent fibrotic mediator, the knockout of which does not experience fibrosis in certain mouse models.^56^ Interactions between multiple chemokines and cytokines amplify the inflammatory response and trigger the recruitment and proliferation of fibroblasts.

### 2.4 Micro‐RNA event: dysregulation of micro‐RNAs

Recent studies have suggested that gene regulators like micro‐RNAs (miRNAs) play a significant role at the transcriptional or post‐transcriptional levels in cells exposed to radiation.^57^ MiRNAs consist of a broad range of endogenous short non‐coding RNA sequences, comprised of 16‐22 nucleotides, that regulate gene expression at the post‐transcriptional level by hybridizing to the 3′ untranslated region of various complementary target mRNAs.^58^ There are approximately 150‐200 miRNAs expressed in cardiomyocytes, many of which are dynamically regulated in response to cardiovascular disease, including in response to cardiovascular toxicity after irradiation.^59^ Previous studies have confirmed that miRNAs participate in the pathological processes involved in RIMF, including DNA damage, OS, inflammation, endothelial dysfunction, hypertrophy and fibrosis.^60^ Further, the levels of expression of many miRNAs are affected by irradiation. MiRNA‐15bM is regarded as a regulator of cardiac hypertrophy and fibrosis and acts by inhibiting the TGF‐β signalling pathway. After irradiation, down‐regulation of this pathway is mediated by up‐regulation of sirtuin 4 or by increasing levels of ROS.^61^ Similarly, after chest irradiation, up‐regulated miRNA‐21 in fibrotic cardiac muscles contributes to the development of cardiac hypertrophy and myocardial fibrosis by regulating growth factor secretion, fibroblast survival and the extracellular signal‐regulated kinase (ERK)‐MAPK signalling pathway in cardiac fibroblasts.^62^ miRNA‐21 can also regulate the programmed cell death 4 protein as well as some cardioprotective mediators such as activator protein‐1, heat‐shock protein‐70, endothelial nitric oxide synthase and heat‐shock transcription factor‐1.^63^ MiRNA‐29 appears to be down‐regulated by TGF‐β to regulate cardiac fibrosis. The miRNA‐29 family targets the mRNA of multiple types of elastin, fibrillin and collagen involved in fibrosis (including collagens type I and III). In one study by Slezak et al, an increase of cardiac fibrosis was observed, along with an altered collagen I/collagen III ratio in the irradiated rat myocardium.^64^ Similarly, cardiac‐specific miRNA‐208 has been proven to be essential for cardiomyocyte hypertrophy, fibrosis and the expression of the beta‐myosin heavy chain.^65^ Along with the aforementioned miRNAs, miRNA‐22, miRNA‐24, miRNA‐133, miRNA‐132 and miRNA‐214 are also associated with the occurrence of myocardial infarction or other heart disease. However, the nature of their relationship to RIMF requires further exploration.^66^, ^67^, ^68^

### 2.5 Neurohumoural event: the activation of the cardiac sensory nervous and renin‐angiotensin system

Myocardial loss after irradiation causes progressive renin‐angiotensin‐aldosterone system (RAAS)‐driven remodelling of the myocardium, which leads to end‐stage symptoms and results in sympathetic overactivation.^69^ In animal experiments, low doses of radiation to the heart have been shown to up‐regulate beta‐receptors. Some studies have suggested that the increased sympathetic stimulation serves to compensate for subclinical myocardial injury.^70^ Furthermore, sensory stimulation may promote the expression of nuclear receptors NR4A1/2 related to the injury of cardiomyocytes and enhance RIMF; however, it may also increase the activity of mast cells to prevent cardiac fibrosis and protect against the loss of cardiac function in the rat.^71^ RAS mediators may also be up‐regulated after cardiac irradiation. Aldosterone has been shown to increase the synthesis of collagen I in cardiac fibroblasts and to increase the number of endothelin receptors. According to Zhao et al, histological characteristics of aldosterone‐induced cardiac fibrosis include intense perivascular inflammation and proliferation of cardiac myocytes and fibroblasts.^72^ Moreover, the impact of angiotensin II‐aldosterone on radiation‐induced heart injury may be stronger as the radiation dose increases.

### 2.6 Neuroimmune event: the cardiac sensory nervous system, mast cells, and endothelin system

Neuroimmune interactions have been widely acknowledged as important regulators of tissue homeostasis and injury. Mast cells found near nerve terminals or heart axons can interact with nerves at the cellular and molecular levels in a variety of ways.^73^ On irradiation, the degranulation of mast cells triggered by ROS generation, complement activation, cytokine stimulation or adenosine may lead to the release of several mast cell‐derived fibrogenic mediators, such as TNF‐α, TGF‐β, IL‐4 and PDGFs, which may trigger or amplify the fibrotic response.^74^ In the normal rat myocardium, mast cells express α‐ and β‐adrenergic receptors, a blockade of which can cause an increase in mast cell degranulation and a decrease in collagen deposition. Whether the accumulation of interstitial collagen III in the hearts of mast cell‐deficient rats is more pronounced post‐irradiation is arguable, suggesting that mast cells play a predominantly protective role in RIMF.^75^ Mast cells can activate proteinase‐activated receptor‐2 on the neuron surface by producing nerve growth factor, which affects neuronal growth and function.^74^

Some sensory neuropeptides such as calcitonin gene‐related peptide (CGRP), neuropeptide Y and substance P have the capacity to induce or enhance the degranulation of mast cells.^76^ Substance P can enhance the secretions of histamine, TNF‐α and TGF‐β1 to amplify the pro‐inflammatory and pro‐fibrotic responses of mast cells to IR. CGRP is an effective vasodilator that benefits the heart through IGF‐1 up‐regulation and TNF‐α down‐regulation, which are both associated with reduced normal tissue radiation injury. It has been shown to have a protective effect in a rat model of radiation enteropathy. However, the role of CGRP in RIMF deserves further investigation.

Similarly, endothelin‐1 (ET‐1), a 21‐amino acid peptide, was initially found to be a potent vasoconstrictor, but also has pro‐inflammatory and pro‐fibrotic properties. Proteinases derived from mast cells have been proven to contribute to both the formation and degradation of ET‐1.^77^ Mast cells expressing the receptor ETA can induce mast cell degranulation on activation by ET‐1; this mechanism allows ET‐1 to improve MMP activity in the heart.^78^

### 2.7 Fibrotic event: abnormal myocardial remodelling

The transdifferentiation of cardiac fibroblasts into contractile and secreted myofibroblasts is the hallmark cellular event driving reparative and fibrotic processes after radiation exposure.^8^, ^79^, ^80^, ^81^ The signalling mechanisms of this process are not yet fully elucidated. Nevertheless, TGF‐β1 is considered one of the most effective initiators for inducing transdifferentiation of myofibroblasts from resident fibroblasts and bone marrow progenitors.^82^ Previous studies have shown that TGF‐β1 is consistently up‐regulated both at the protein and the mRNA level in cardiac fibrosis and remodelling.^78^ The canonical pathway of TGF‐β1 signalling is the main axis, where TGF‐β1 first binds heterodimeric receptors in the plasma membrane composed of the TGF‐β type I and type II half‐receptors before inducing phosphorylation of Smad2 and Smad3 transcription factors, complexed with Smad4, in the cytoplasm. Subsequently, the transcription factors transfer to the nucleus and form a transcriptional complex, transactivating key ECM genes such as those that encode type I collagen.^82^ Matricellular protein CTGF is considered a downstream mediator of the TGF‐β1‐induced activation of fibroblasts. In addition, CTGF can also be induced through the activation of the Rho/ROCK pathway independently of TGF‐β1/Smad signalling.^83^ Alongside the Smad‐mediated pathways, TGF‐β receptor activation can also initiate non‐canonical signalling to activate the p42/p44 MAPK cascade in fibroblasts, stimulating myofibroblast conversion through signals involving the signalling effector calcineurin and some transcription factors.^84^ Activated myofibroblasts can enhance the synthesis of ECM protein (consisting of a complex assortment of collagens, glycoproteins, proteoglycans and matricellular proteins), increase the expression of integrins (overactive tissue inhibitors of matrix metalloproteinases) and suppress MMPs.^81^ Moreover, damaged endocardial endothelium and epicardial epithelium may undergo either an endothelial‐mesenchymal (EndMT) or an EMT regulated by TGF‐β1 to acquire phenotypic characteristics of a myofibroblast such as type I collagen secretion and α‐SMA expression.^85^, ^86^ The direct expression of proto‐oncogenes such as c‐Jun and c‐Myc probably also contributes to late fibrotic changes.^8^ Abnormal accumulation of ECM separating and/or replacing myocytes increases ventricular stiffness, decreases elasticity and distensibility and can lead to contractile dysfunction.^87^ Furthermore, excess fibroblasts and ECM can cause damage to the mechano‐electric coupling of cardiomyocytes, thus decreasing cardiac contraction and raising the risk of arrhythmogenesis and mortality. Besides, inflammation and fibrosis within perivascular regions may cause a reduction in tissue utilization of oxygen and nutrients and increase the adverse cardiac remodelling.^88^ These factors ultimately lead to decreased elasticity and distensibility, thereby resulting in reduced ejection fraction and cardiac failure.^2^

## 3 THERAPEUTIC STRATEGIES

From the perspective of radioactive protection, the prevention of RIMF is more important than its treatment. The understanding and management of conventional cardiovascular risk factors, such as hypertension and hypercholesterolaemia, have great significance to improve prognosis. Studies have shown that conventional risk factors are the main indicators for the development of cardiovascular disease after irradiation.^89^ Improvements to RT techniques, such as 3‐dimensional treatment planning, intensity‐modulated RT, stereotactic body RT, image‐guided therapy, deep inspiration breath‐hold, and respiratory gating techniques, will further improve targeting, minimize cardiac exposure to RT and will likely reduce RIMF. However, the risks associated with current techniques are still uncertain as the heart inevitably receives radiation doses during RT and there remains a lack of evidence indicating reliable dose‐response relationships for these therapies. Therefore, additional therapeutic strategies are needed to control fibrosis. (Table 1).

**TABLE 1:** Summary of the effect of different drug classes on RIMF

Category	Name	Molecular type	Target/action	Stage of project trial	Researcher and year (Ref.)
Antioxidants and anti‐inflammatory agents	Amifostine	Ammonia thiol Compound	Scavenging ROS and free radicals	In clinical use	Gurses et al^91^
	Tocomin suprabio	Vitamin E analogs	Inhibiting radiation‐induced dysfunction of cardiac mitochondria	Preclinical	Sridharan et al^93^
	Pentoxifylline and α‐tocopherol	Antioxidant mix	Migrating Inflammatory cells, scavenging free radicals, and down‐regulating the level of TGF‐β1	Preclinical	Liu et al^94^
	Melatonin	N‐acetyl‐5‐methoxytryptamine	Suppressing IL‐4 and pro‐oxidant enzymes including NOX2, NOX4, and Duox1/Duox2	Preclinical	Gurses et al^98^
	Metformin	A lipophilic biguanide	Attenuating up‐regulation of NOX4 and Duox1/Duox2, and decreasing the infiltration of lymphocytes and macrophages	Preclinical	Karam et al^100^
	Selenium‐L‐methionine	Selenium analogs	Decreasing IL‐4 and Duox1/Duox2	Preclinical	Kolivand et al^40^
	Resveratrol	3,4,5‐trihydroxy‐trans‐stilbene	Alleviating oxidative stress, inflammation responses, and expression of TGF‐β1	Preclinical	Wu et al^103^
	Colchicine	Tricyclic alkaloid	Inhibiting microtubule polymerization and reducing platelet aggregation	Preclinical	O'Herron et al^96^
	CAPE	A bioactive compound of propolis extract	Antioxidant and anti‐inflammatory	Preclinical	Mansour et al^97^
	L‐carnitine	L‐3‐hydroxy‐4‐N‐N‐N‐trimethylaminobutyrate	Activating ROS/p38 MAPK signalling	Preclinical	Fan et al^104^
Statins	Pitavastatin	HMG‐coA inhibitor	Inhibiting the Rho‐ERK signalling pathway	Preclinical	Saka et al^107^
	Simvastatin	HMG‐coA inhibitor	Reducing the proliferation of atrial myofibroblasts	Preclinical	Saka et al^107^
	Atorvastatin	HMG‐coA inhibitor	Reducing the mRNA and protein Levels of TGF‐β	Preclinical	Zhang et al^108^
	Pravastatin	HMG‐coA inhibitor	Decreasing expression of CTGF, TGF‐β1 and Collagen I alpha2	Preclinical	Doi et al^109^
ACEIs	Captopril	ACE inhibitor	Reducing myocardial perivascular fibrosis and myocardial cell apoptosis	Preclinical	Ong et al^113^
Targeted therapies	IPW‐5371	A monoclonal antibody	Inhibitor of TGF‐β receptor 1	Preclinical	Rabender et al^114^
	Rhnrg‐1β	An epidermal growth factor ‐like protein	Alleviating early mitochondrial dysfunction and late myocardial fibrosis and dysfunction	Preclinical	Gu et al^115^
	Antagomir‐21	Mir‐21 inhibitors	Silencing mir‐21	Preclinical	Thum et al^117^
Stem cell therapy	Msc	Stem cell mobilizer	Trans‐differentiating and secreting high levels of paracrine factors	Preclinical	Gao et al^121^
Traditional Chinese medicine	Shensongyangxin	Chinese herb	Down‐regulating the mrna levels of TGF‐β1 and mmps, and decreasing the protein levels of Col I and Col III	Preclinical	Ma et al^126^
	Aristolochia yunnanensis	Chinese herb	Regulating ERK1/2 and TGF‐β/Smad pathways	Preclinical	Shao et al^127^
	Tanshinone IIA	Chinese herb	Down‐regulating the levels of TGF‐β1 and NF‐κB p65.	Preclinical	Ma et al^80^
	Puerarin	Chinese herb	Decreasing the expression of NF‐κB and TGF‐β1	Preclinical	Chen et al^128^

### 3.1 Antioxidants and anti‐inflammatory agents

As discussed above, the interaction between OS and the inflammatory response induced by IR is a major process in the development of RIMF. Antioxidant and anti‐inflammatory therapies are effective measures to prevent and even reverse RIMF. Amifostine, a thiol‐containing free radical scavenger, is currently the only FDA‐approved radioprotector and has been shown to have potential therapeutic effects by inhibiting OS, diminishing the number and severity of myocyte necrosis and delaying myocardial fibrosis.^90^ Studies also suggest that the administration of amifostine prior to irradiation could significantly improve coronary flow, decrease vasculitis and vascular damage associated with fibrosis development. However, amifostine is seldom applied to clinical therapy because of its severe side effects (nausea, vomiting and diarrhoea).^91^ Tocotrienols, the natural vitamin E analogs, are emerging as the strongest radioprotector of all natural compounds tested to date and may sensitize cancer cells to radiation and have the capacity of anti‐cancers.^92^ Pre‐treatment with Tocomin SupraBio (TSB), a tocotrienol‐enriched preparation, could inhibit radiation‐induced changes in cardiac mitochondria, including susceptibility to mPTP opening, decreased mitochondrial respiration and reduced mitochondrial membrane potential. However, TSB pre‐treatment could not prevent long‐term cardiac function alterations or adverse cardiac remodelling induced by radiation.^93^ Furthermore, a pentoxifylline (PTX) and α‐tocopherol combination could down‐regulate the expression of TGF‐β1 mRNA and modify the development of myocardial fibrosis in the irradiated hearts of rats.^94^ Whether started before irradiation or during the process of RIMF, the combination of PTX and α‐tocopherol may improve left ventricular diastolic dysfunction and benefit RIMF. However, oral administration of PTX alone 3‐6 months after RT did not obviously ameliorate intracellular signalling or adverse myocardial remodelling, but instead had adverse effects on cardiac rhythm of local irradiated heart in one instance.^95^

In addition to the above drugs, colchicine exerts its anti‐inflammatory and anti‐coagulant properties through the inhibition of microtubule polymerization and reduction of platelet aggregation, preventing coronary artery disease induced by radiation.^96^ Heba et al have shown that caffeic acid phenethyl ester (CAPE) could alleviate cardiac‐oxidative impairment induced by radiation in rats, which significantly reduces the activities of xanthine oxidase, adenosine deaminase, and malondialdehyde and increases the levels of total SOD and nitrate/nitrite in the heart tissue.^97^ Melatonin (N‐acetyl‐5‐methoxytryptamine), the chief product of the pineal gland‐Q, can prevent vasculitis, and decrease necrosis and fibrosis of cardiomyocytes by suppression of some cytokines such as IL‐4 and pro‐oxidant enzymes like NOX2, NOX4 and Duox1/Duox2 in radiation‐induced heart injury rat models.^98^ Metformin, widely recognized as an anti‐diabetic drug, has been shown to have a certain degree of radioprotective effects including anti‐oxidation and anti‐fibrosis.^41^, ^99^ It has been confirmed that metformin potently can attenuate up‐regulation of TGF‐β–NOX4 pathway and DUOX1/DUOX2, and decrease the infiltration of inflammatory cells such as lymphocytes and macrophages to protect against heart and lung injury in irradiated rat models.^42^, ^100^, ^101^ Mouse experiment has shown that that selenium‐L‐methionine can protect against radiation‐induced injury to tissues of heart and lung by the down‐regulation of pro‐oxidant enzymes like Duox1 and Duox2 and modulation of some cytokines like IL‐4.^40^ Resveratrol which is a natural polyphenol has been shown to significantly decrease cardiac fibroblast proliferation and collagen secretion by alleviating OS, inflammation responses and the expression of TGF‐β1.^102^, ^103^ Recent investigations have noted that L‐carnitine suppressed intracellular ROS accumulation and myocyte apoptosis in radiation‐exposed hearts to exert cardioprotective effects, the mechanisms of which involved ROS/p38 MAPK signalling. Activated p38 MAPK promotes the separation of NRF2 from KEAP1, thus initiating the transcription of a variety of antioxidant and anti‐apoptotic genes, such as quinine oxidoreductase‐1 and haem oxygenase‐1.^104^

### 3.2 Statins

Statins, the 3‐hydroxy 3‐methylglutaryl‐CoA reductase (HMG‐CoA) inhibitors, lower serum cholesterol and lipoprotein density in plasma to decrease cardiac morbidity and mortality.^105^ Increasing evidence suggests that statins may be interesting agents for radiomitigation as they exhibit antioxidant properties, improve endothelial dysfunction, inhibit inflammatory responses and suppress collagen fibre production.^106^ Several animal experiments have shown that statins can attenuate cardiac fibrosis. Saka et al have reported that pitavastatin inhibits the Rho‐ERK‐serum response factor signalling pathway to attenuate cardiac hypertrophy and fibrosis, independently of its cholesterol‐lowering action.^107^ Simvastatin has been shown to reduce the proliferation of atrial myofibroblasts, independent of cholesterol synthesis, via inhibition of RhoA in vitro.^107^ In another study by Zhang et al, atorvastatin reduced the mRNA and protein levels of TGF‐β, reduced fibronectin expression and ultimately ameliorated RIMF in rats; these effects were especially apparent in trials with longer and higher doses of treatment.^108^ The proposed mechanism was through the regulation of the TGF‐β/Smad3, Rho/ROCK and PI3K/Akt signalling pathways. Similarly, pravastatin decreases the expression of CTGF, TGF‐β1 and COL1A2 to inhibit radiation‐induced tissue fibrosis, although the effect of irradiation on the tumour is preserved.^109^

### 3.3 Angiotensin‐converting enzyme inhibitors (ACEIs)

It is known that the RAAS plays an important pathophysiological role in cardiac fibrosis and remodelling after irradiation exposure, the influence of which becomes worse with increasing radiation dose.^110^ ACEIs involved in RAAS have been widely applied to the management of congestive heart failure and hypertension.^111^ A substantial body of evidence suggests that the ACEI captopril may ameliorate radiation‐induced toxicity in organs such as the central nervous system, lungs and heart.^112^ Captopril not only inhibits inflammatory responses and the production of ROS but also increases the production of NO and prostacyclin for cardioprotection. This occurs through the suppression of bradykinin breakdown after simultaneous radiation of the heart and lungs, thereby reducing myocardial perivascular fibrosis and myocardial cell apoptosis.^113^ Though captopril acts to prevent changes in capillary function, nerve terminals, mast cells, atrial granule numbers and fibrosis, it does not prevent the progressive deterioration of cardiac function after irradiation. However, these effects are thought to be caused by the properties of captopril instead of by ACEIs.^111^ The role of ACEIs in a potential intervention for cardiac radiation injury requires further investigation.

### 3.4 Molecular targeted therapies

The critical role of TGF‐β as a post‐irradiation fibrosis driver highlights its potential as an antifibrotic agent and radiation countermeasure in the treatment of RIMF. In one study by Christopher et al, IPW‐5371, a small molecule inhibitor of TGF‐β receptor 1, significantly decreased the level of TGF‐β‐induced phosphor‐Smad3; this resulted in decreased deposition of collagen in the heart/lungs and evidently improved cardiopulmonary function in irradiated mice. More studies to determine non‐TGF‐β‐mediated Smad2/3 activity and non‐Smad2/3 TGF‐β activity like MAPK, PI3K, JNK and Rho proteins are required to fully clarify the mechanism of IPW‐5371 in reducing fibrosis.^114^

Another animal experiment revealed that the epidermal growth factor (EGF)‐like protein rhNRG‐1β effectively reduced radiation‐induced myocardial nuclear injury, maintained the homeostasis of mitochondria in cardiomyocytes after radiation, mediating long‐term protective effects on cardiac pump function, myocardial metabolism and myocardial tissue structure. These protective effects are exerted, at least in part, by the transduction of ErbB2‐ERK‐SIRT1 pathway.^115^ Because rhNRG‐1β is a promising factor for the therapy of heart injury after irradiation, its potential to promote tumour growth must be considered.^116^

In addition to these considerations, miRNAs have been shown to modulate multiple cardiac functions and may also be promising therapeutic targets. In one mouse pressure‐overload‐induced disease model, antagomir‐21 silencing miR‐21 could suppress the activity of cardiac ERK–MAPK, reduce interstitial fibrosis and alleviate cardiac dysfunction.^117^ However, miRNA‐based therapy is limited, as treatment targets more than one gene during application and may influence other pathways in the organism.^118^

### 3.5 Stem cell therapy

In the past 20 years, abundant adult stem cells have already been isolated and identified for application to cardiac cell therapy in the basic science and clinical fields.^119^ Although the mechanism of action is not fully understood, there is a general consensus that stem cell therapy has the ability to benefit heart function post‐injury and inhibit adverse remodelling.^120^ Transplanted stem cells are able to trans‐differentiate into vascular cells and cardiomyocytes to repair injured cardiac tissues. However, the above mechanisms failed to exactly explain for overall improvements in cardiac remodelling and function, due to the poor retention and low survival of transplanted stem cells.^120^ The widely accepted potential mechanism is that stem cells have capacity of secreting high‐level paracrine factors that enhance endogenous reparative and regenerative processes, including inhibiting cell apoptosis, mediating the existing cardiomyocytes survival, promoting endogenous stem cell populations activation and homing to the damaged site, modulating inflammation, promoting new blood vessel formation, improving cardiac hypertrophy, favourably changing the ECM and preventing fibrosis.^119^ Animal models have indicated that mesenchymal stem cells（MSCs）extracted from bone marrow transplantation can significantly improve cardiac functions, alleviate inflammatory reactions and myocardial fibrosis, and recruit DNA repair proteins to facilitate DNA repair after irradiation.^121^ However, factors such as the optimal cell type, number of cells, timing of therapy and route of administration for the application of stem cell therapy remain unclear.^122^ Human‐induced pluripotent stem cells differentiate into beating cardiac myocytes when exposed to an average absorbed dose of < 10 Gy; with increasing radiation doses, the differentiation ability of pluripotent stem cells declines.^123^ Some investigators suggest that MSC‐induced immune cell dysfunction, immune suppression, angiogenesis and cell division can accelerate tumour progression.^124^ Pluripotent stem cells commonly acquire TP53 mutations before increasing cancer formation risk.^125^ Therefore, these issues should be addressed via carefully planned preclinical/clinical trials before stem cell therapy can be routinely and safely administered for RIMF.

### 3.6 Traditional Chinese medicine

With technological and biological advances in the clinical field, experimental studies and clinical evaluations have indicated that the use of Chinese herb‐derived compounds is an effective method for treating myocardial fibrosis. Chinese herbs have been proven to have anti‐oxidation, anti‐inflammation, anti‐apoptosis, anti‐fibrosis, pro‐angiogenesis and regulatory metabolic properties. An experiment utilizing a rabbit model demonstrated that the shensongyangxin capsule could down‐regulate the mRNA levels of MMP‐2, MMP‐9, TIMP‐I and TGF‐β1 and decrease the protein levels of type I and III collagen, thereby ameliorating electrophysiological dysregulations in the ischaemic heart and inhibiting the differentiation of cardiac fibroblasts to myofibroblasts.^126^ *Aristolochia yunnanensis* can inhibit cardiac fibrosis induced by angiotensin II via ERK1/2 and TGF‐β/Smad signalling pathways.^127^ Chang et al found that tanshinone IIA alleviated myocardial fibrosis in pressure‐overloaded rats, the mechanisms of which might be associated with the inhibition of the expression of Rho‐associated coiled‐coil protein enzyme 1 and the down‐regulation of NF‐κB, p65 and TGF‐β1.^80^ Similarly, puerarin effectively decreases the expression of NF‐κB and TGF‐β1 and plays a role in the prevention of myocardial fibrosis.^128^

## 4 CONCLUSION

Although modern radiotherapy protocols have reduced the radiation exposure of non‐target cardiovascular structures in the past few years, RIMF is still a growing concern for cancer survivors. It is essential to identify predictive biomarkers that contribute to select susceptible patients to reduce radiation dosage and avoid the occurrence of RIMF. Preventive measures should be actively taken to reduce aggressive risk factors according to published guidelines. A variety of signalling pathways involving RIMF indicate potential targets for intervention strategies. Routine clinical approaches for RIMF have not been approved, although urgent treatments are needed. There are many obstacles and unknowns with any novel therapeutic in the research and development phases; therefore, a considerable number of preclinical and clinical studies are needed to evaluate the safety, optimal dosage, time and route of administration.

## CONFLICT OF INTEREST

The authors declare no conflict of interest.

## AUTHOR CONTRIBUTIONS

XJ and YX: Conceptualization. BW and HHW: software; investigation. MMZ: resources. BW, HHW, and JLW: writing‐original draft preparation. RJ, YX, and XJ: writing‐review and editing. X.J: funding acquisition. All authors read and approved the manuscript.
